# Rapid and sensitive detection of Senecavirus A by reverse transcription loop-mediated isothermal amplification combined with a lateral flow dipstick method

**DOI:** 10.1371/journal.pone.0216245

**Published:** 2019-05-02

**Authors:** Jinhui Li, Weifang Liang, Shuaifei Xu, Jian Shi, Xia Zhou, Bowen Liu, Li Yu, Jingfeng Xiong, Guangbin Si, Dongsheng He

**Affiliations:** 1 College of Veterinary Medicine, South China Agricultural University, Guangzhou, China; 2 Key Laboratory of Zoonosis Prevention and Control of Guangdong Province, Guangzhou, China; 3 Key Laboratory of Comprehensive Prevention and Control for Severe Clinical Animal Diseases of Guangdong Province, Guangzhou, China; University of Georgia, UNITED STATES

## Abstract

Senecavirus A (SVA) is a critical pathogen causing vesicular lesions in sows and acute death of newborn piglets, resulting in very large economic losses in the pig industry. To restrict the transmission of SVA, an establishment of an effective diagnostic method is crucial for the prevention and control of the disease. However, traditional detection methods often have many drawbacks. In this study, reverse transcription loop-mediated isothermal amplification (RT-LAMP) was combined with a lateral flow dipstick (LFD) to detect SVA. The resulting RT-LAMP-LFD assay was performed at 60°C for 50 min and then directly judged on an LFD visualization strip. This method shows high specificity and sensitivity to SVA. The detection limit of RT-LAMP was 4.56x10^-8^ ng/μL RNA, approximately 11 copies/μL RNA, and it was 10 times more sensitive than RT-PCR. This detection method’s positive rate for clinical samples is comparable to that of RT-PCR. This method is time saving and highly efficient and is thus expected to be used to diagnose SVA infections in this field.

## Introduction

Senecavirus A (SVA), formerly known as Seneca Valley virus (SVV)[1], is the only recognized species of the genus Senecavirus within the family Picornaviridae[2], and it was first isolated in 2002[3]. SVA is clinically characterized by vesicular ulcers in the coronary arteries or nostrils[4]. Therefore, it is difficult to differentiate from foot-and-mouth disease (FMD), swine vesicular disease (SVD), and vesicular stomatitis (VS) in clinical symptoms[5]. SVA is a small, nonenveloped RNA virus with a single sense strand 27–30 nm in diameter. The viral genome is approximately 7.3 kb in length and consists of a 5’ noncoding region (UTR), a 3’ noncoding region (UTR) and a large open reading frame[3]. The only open reading frame encodes a polymeric precursor protein including an L leader protein, a P1 region structural protein, and the P2 and P3 region nonstructural proteins, which is very similar to other members of the small RNA virus family (Picornaviridae)[2, 3]. From the 5' to 3' direction, the genomic structure L-VP4-VP2-VP3-VP1-2A-2B-2C-3A-3B-3C-3D is the same as that of the picornavirus L-4-3-4 genome structure[3].

SVA is clinically difficult to distinguish from other classic viral vesicular diseases[6], which increases the difficulty of clinical diagnosis. Therefore, it is necessary to establish a series of laboratory diagnostic methods for the diagnosis and identification of vesicular diseases. Reverse transcription droplet digital PCR assay (RT-ddPCR) [7], Indirect immunofluorescence assay (IFA)[8], reverse transcription polymerase chain reaction (RT-PCR)[9, 10], reverse transcription quantitative PCR (qRT-PCR)[11], novel RNA-based in situ hybridization[12] and enzyme-linked immunosorbent assays (ELISAs)[13] have also been applied to detect SVA. These methods show high specificity and sensitivity but place high demands on experimental instruments and the skills of research personnel. Therefore, the above methods are not suitable for laboratories with poor clinical and experimental equipment.

Loop-mediated isothermal amplification (LAMP) is a new PCR-based molecular amplification method invented by Notomi in 2000[14]. It can efficiently, rapidly and specifically amplify the target sequence under isothermal conditions and relies on primers that have the ability to recognize six specific regions on the target sequence, a Bst DNA polymerase with a helicase function and LAMP of the target sequence[14–17]. LAMP techniques have high specificity, sensitivity, and stability, and studies have shown that LAMP has similar sensitivity as ordinary PCR[18–20]. LAMP can detect RNA templates by using reverse transcriptase[14, 21], and the RT-LAMP method has been applied to the detection of multiple viruses[18, 22–24]. Amplification by this method can occur in a common water bath, and the amplified product can be visually identified by adding a fluorescent dye[25]. The method is suitable for the detection of a primary layer and the clinical site.

The lateral flow dipstick (LFD) method utilizes a biotin-labeled LAMP amplification product to specifically hybridize to a probe labeled with fluorescein isothiocyanate (FITC), which thereby binds to a colloidal gold-labeled anti-FITC antibody to form a ternary complex that is bound at a biotin-containing detection line, while unhybridized FITC-labeled probe forms a biotin-free binary complex and is bound at an anti-FITC quality control line[26]. Recently, an RT-LAMP-LFD assay has been successfully used for the detection of a variety of viral pathogens[27–30]. The method has the advantages of high specificity, high sensitivity, good stability and repeatability. In addition, the RT-LAMP-LFD assay avoids false positives caused by agarose gel electrophoresis or fluorescent dye staining[31].

In this study, LAMP primers were designed to target the conserved regions of the SVA 3D gene sequence. An SVA RT-LAMP method was established. We combined RT-LAMP with a LFD visualization strip to develop an RT-LAMP-LFD assay that facilitates the diagnosis of SVA. The method has excellent specificity, high sensitivity, and good stability and repeatability and provides a reliable method for preliminary screening and detection of SVA in ordinary laboratories and at sites in the field.

## Materials and methods

### Viruses

Senecavirus A (SVA; HN16 strain, Accession number: MF893200), porcine deltacoronavirus (PDCoV; PDCoV/CHGD/2016 strain, Accession number: MH715491), porcine epidemic diarrhea virus (PEDV; CH/GDGZ/2012 strain, Accession number: KF384500), porcine transmissible gastroenteritis virus (TGEV; CN12 strain, Accession number: KX058075), and porcine reproductive and respiratory syndrome virus (PRRSV; JXA1 strain, Accession number: EF112445) were obtained from the virus repository of the College of Veterinary Medicine of South China Agricultural University. A foot-and-mouth disease virus (FMDV) vaccine strain and classical swine fever virus (CSFV) standard positive serum were purchased from the Beijing Century Yuanheng Animal Epidemic Prevention Technology Co., Ltd. The whole genome of swine vesicular disease virus (SVDV; HK'70 strain, Accession number: AY429470) and vesicular stomatitis virus (VSV; Arizona/5481555/2015 strain, Accession number: KT429217) was synthesized by Beijing Qingke Biotechnology Co., Ltd.

### Clinical sample collection and testing

From September to December 2017, 33 clinical serum samples were collected from different suspected pig herds in Guangzhou, Guangdong Province. Samples were collected from weaned piglets and sows. Blood samples were collected from the ear-rim auricular vein using a 5ml disposable sterile syringe. Blood sample of 4 ml was collected from each suspected infected pig. The collected blood was placed in a 5 ml sterile centrifuge tube and allowed to stand at room temperature for 30 min. Serum samples were obtained by centrifugation at 1200 g for 10 min. All serum samples were shipped at 0°C and stored at -80°C until total RNA was isolated. The sampling procedure was approved by the Animal Ethics Committee of Guangdong Province, China. The sampling process was assisted by local authorities and veterinarians. Samples of the disease were obtained after veterinary pathological examination.

### Nucleic acid extraction

Viral RNA of SVA (HN16 strain), PDCoV (PDCoV/CHGD/2016 strain), PEDV (CH/GDGZ/2012), TGEV (CN12 strain), and PRRSV (YA strain) were extracted from 300 μL of infected cell culture supernatant. Meanwhile, viral RNA of the above viruses, the FMDV vaccine strain and classical swine fever virus (i.e., CSFV) standard positive serum were extracted using TRIzol reagent (TaKaRa Biotechnology, Dalian, China) according to the manufacturer's instructions. In addition, 300 μL of each clinical serum sample was subjected to RNA extraction using TRIzol reagent. The extracted RNA was resuspended in 50 μL of RNase-free water (Gibco BRL, USA) and stored at -80° C until further testing. Positive plasmids of SVDV and VSV were extracted by Plasmid Mini Kit I (200) (Omega Bio-tek, Norcross, GA, USA).

### Primers

**Fig 1:**
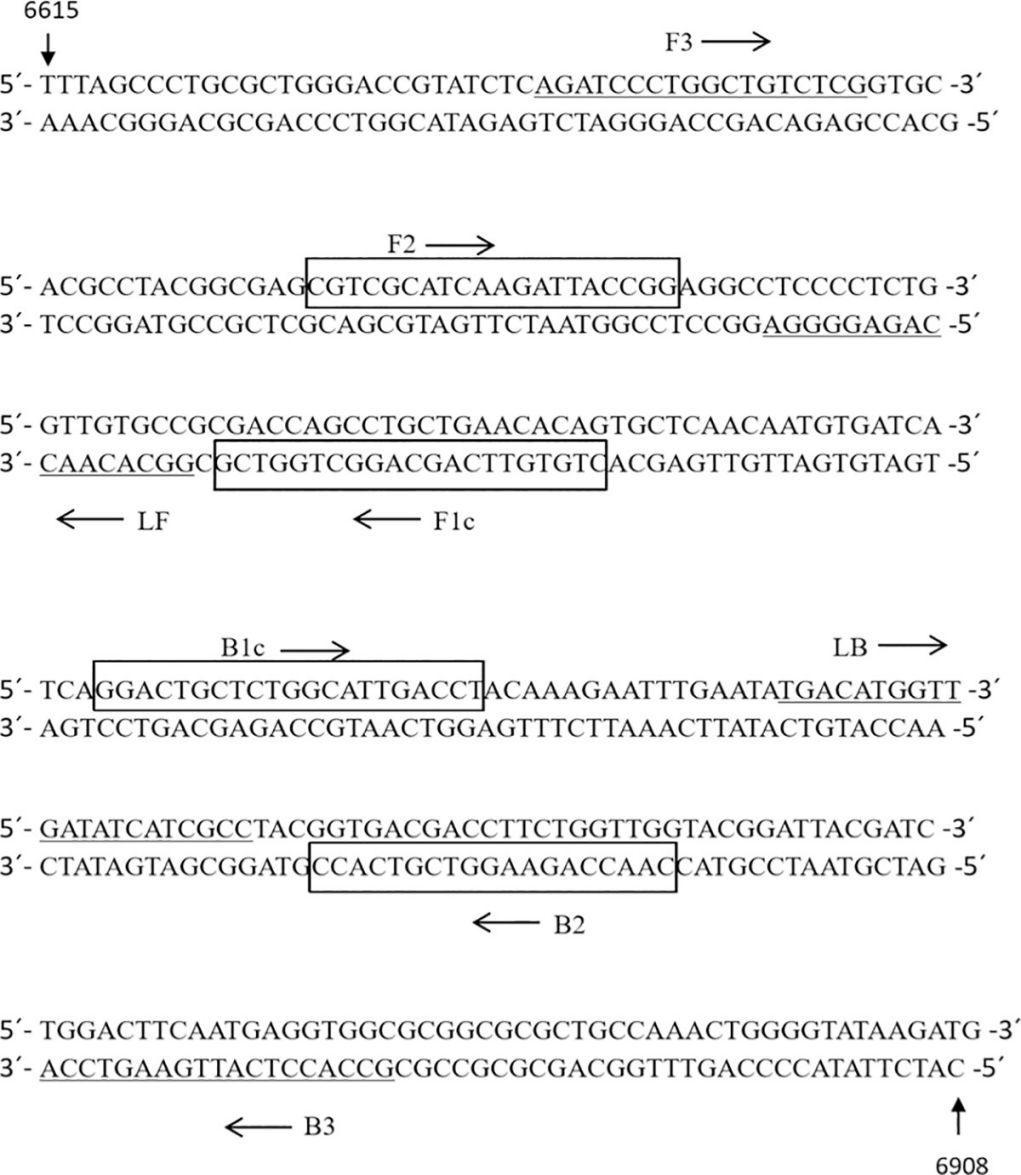
Schematic showing location of RT-LAMP primer binding sites within SVA 3D gene. SVA RT-LAMP primer binding sites. The assay spans nucleotides 6615–6908 with reference to the 3D gene sequence of the SVA virus strain SVA-HN16.

Thirty-one full-length sequences of SVA were obtained from GenBank(GenBank Accession numbers: MF893200, MK256736, NC-011349, KR063107, KT321458, KT757282, KU359210, KU359214, KU954090, KX223836, KX857728, KY038016, KY419132, KY486163, KY618837, KY747510, KY747511, KY747512, MF416220, MF967574, MG428680, MG428681, MG428682, MG428683, MG428684, MG428685, MG765550, MG765552, MH316114, MH704432) and aligned by MegAlign (Larsergene, version 7.0), and it was found that its 3D region was more conservative (S1 Fig). LAMP primers were designed based on the conserved regions of the SVA 3D gene sequence. RT-LAMP primer-assisted design software Primer Explorer V5 (http://primerexplorer.jp/e/) was used to design the RT-LAMP primers of the SVA gene based on the determined conserved nucleotide sequence (HN16 strain, GenBank Accession number MF893200). The RT-LAMP primer sets for detecting SVA includes a forward primer (SVA-FIP), a reverse internal primer (SVA-BIP), a forward external primer (SVA-F3), a reverse external primer (SVA-B3), a forward loop primer (SVA-LF) and a reverse loop primer (SVA-LB). The FIP primer was labeled with biotin at the 5' end, and the BIP primer was labeled with FITC at the 5' end (Table 1). In addition, the conserved region of the SVA VP1 gene sequence was used in the design of common RT-PCR detection primers (Table 2). The synthesis and labeling of all primers was done by the BGI TECH SOLUTIONS (BEIJING LIUHE Co., Ltd, Beijing, China) (Fig 1).

**Table 1:** Details of primers used for RT-LAMP assay.

Name	Type	Position	Sequence (5´→3´)
SVA-F3	Forward outer	6642–6659	5´-AGATCCCTGGCTGTCTCG-3´
SVA-B3	Reverse outer	6859–6877	5´-GCCACCTCATTGAAGTCCA-3´
SVA-FIP^a^(F1c+F2)	Forward inner	F1c: 6721–6741	5´-CTGTGTTCAGCAGGCTGGTCG -CGTCGCATCAAGATTACCGG-3´
		F2: 6678–6697	
SVA-BIP^b^(B1c+B2)	Reverse inner	B1c: 6763–6783	5´-GGACTGCTCTGGCATTGACCT -CAACCAGAAGGTCGTCACC-3´
		B2: 6825–6843	
SVA-LF	Forward loop	6703–6719	5´-GGCACAACCAGAGGGGA-3´
SVA-LB	Reverse loop	6800–6821	5´-TGACATGGTTGATATCATCGCC-3´

a 5´-Labeled with biotin when used in RT-LAMP-LFD assay.

b 5´-Labeled with FITC when used in RT-LAMP-LFD assay.

**Table 2:** Details of primers used for RT-PCR assay.

Name	Type	Position	Sequence (5´→3´)
SVA-F	Forward	2542–2564	5´-ATGGTTGGTTTAGCCTGCACAAG-3´
SVA-R	Reverse	3260–3284	5´-AAGCACGGATGAGACAGAGTTCCAA-3´

### Optimization of reaction conditions for loop-mediated isothermal amplification

#### Establishment of initial RT-LAMP-LFD detection method

The RT-LAMP assay was carried out in a 25 μL reaction mixture system containing 10×ThermoPol Buffer (New England Bio-labs, USA), 2.5 mM dNTPs (TaKaRa Biotechnology, Dalian, China), 10 μM SVA-FIP and SVA-BIP, 10 μM SVA-LF and SVA-LB, 10 μM SVA-F3 and SVA-B3, 100 mM MgSO_4_ (New England Bio-labs, USA), 8 U/μL Bst DNA polymerase (8U, New England Bio-labs, USA), 5 U/μL AMV reverse transcriptase (5 U, TaKaRa Biotechnology, Dalian, China) and 3 μL (1.368ng) of RNA was used as a template or 3 μL of ddH_2_O as a template for a negative control. The amplification was incubated for 60 min in a constant temperature water bath at 60°C, then the reaction was terminated by raising the temperature to 80°C for 4 min. Subsequently, the reaction product in the reaction tube was used for LFD (Ustar Biotech, Hangzhou, China) visual test strip detection. The tested samples included a negative control without a template. After 10–15 min, the results were observed by eye and recorded. If both the T line and the C line appeared, the result was a positive amplification. If only the C line was displayed, the result was a negative amplification. When both the T line and C line were not visible, the LFD was considered invalid. The RT-LAMP products were also routinely analyzed by calcein (Beijing Solarbio Science & Technology Co., Ltd.) RT-LAMP assay and 1.2% agarose gel (Gibco BRL, USA) electrophoresis to verify the expected results of positive LAMP reaction products. Gel electrophoresis was performed in 1×TAE buffer (Gibco BRL, USA) at 120V for 35 minutes.

#### Optimization of SVA RT-LAMP reaction conditions

To determine the optimal RT-LAMP reaction conditions, the temperature and time of the RT-LAM reaction were optimized. The RT-LAMP reaction was carried out with the 8 temperatures 57°C, 58°C, 59°C, 60°C, 61°C, 62°C, 63°C and 64°C. Reaction products were analyzed by electrophoresis on 1.2% agarose gels, and the optimal reaction temperature was determined by the amplification effect. RT-LAMP reactions were carried out for the 8 times of 30 min, 35 min, 40 min, 45 min, 50 min, 55 min, 60 min and 65 min. The reaction products were analyzed by electrophoresis on 1.2% agarose gels, and the optimal reaction time was determined by the amplification effect.

#### Optimization of RT-LAMP reaction system

To determine the optimal RT-LAMP reaction conditions, the RT-LAM reaction system was optimized. The 6 Mg2+ concentrations 0 mM, 2 mM, 4 mM, 6 mM, 8 mM and 10 mM were used for the RT-LAMP reaction, and the remaining components were unchanged. The optimal Mg2+ concentration was determined according to the amplification effect. RT-LAMP reactions were carried out with the 8 dNTP concentrations 0.2 mM, 0.25 mM, 0.3 mM, 0.35 mM, 0.4 mM, 0.45 mM, 0.5 mM and 0.55 mM, and these experiments were carried out under optimized conditions and component concentrations. The optimal dNTP concentration was determined by the amplification effect. The 6 Bst DNA polymerase concentrations 0.08 U/μL, 0.16 U/μL, 0.24 U/μL, 0.32 U/μL, 0.4 U/μL and 0.48 U/μL were used for RT-LAMP reactions with optimized conditions and compositions, and these experiments were performed to determine the optimal Bst DNA Polymerase concentration based on the amplification effect. The 6 AMV concentrations 0.02 U/μL, 0.04 U/μL, 0.06 U/μL, 0.08 U/μL, 0.1 U/μL, and 0.12 U/μL were used for RT-LAMP reactions, and these experiments were performed with optimized conditions and compositions. The optimal AMV concentration is determined by the amplification effect. The ratio between the primers SVA-LF and SVA-LB was selected as a reference, and 8 concentration ratios of SVA-FIP and SVA-BIP (2:1, 3:1, 4:1, 5:1, 6:1, 7:1, 8:1, 9:1) were tested with optimized conditions and compositions. The optimal concentration of SVA-FIP and SVA-BIP was determined by the amplification effect. In addition, the ratio of the primers SVA-FIP and SVA-BIP was picked as a reference, and 7 concentration ratios of SVA-LF and SVA-LB (0:1, 1:1, 2:1, 3:1, 4:1, 5: 1, 6:1) were tested with optimized conditions and compositions. Finally, the optimal concentration of SVA-LF and SVA-LB was determined by the amplification effect.

### Specificity of SVA RT-LAMP-LFD

According to the optimized RT-LAMP reaction conditions, The viral RNA extracted from SVA (HN16 strain), PDCoV (PDCoV/CHGD/2016 strain), PEDV (CH/GDGZ/2012 strain), TGEV (CN12 strain), PRRSV (JXA1 strain), the FMDV vaccine strain), the CSFV standard positive serum and positive plasmids of SVDV and VSV were used as templates to test the specificity of the method. The reaction products were analyzed by LFD detection, calcein RT-LAMP assay and 1.2% agarose gel electrophoresis. Each test was repeated at least 3 times.

### Sensitivity of SVA RT-LAMP-LFD

SVA standard template (45.6 ng/μl) were prepared from genomic total RNA extracted from 300 μl of SVA-infected PK-15 cell line culture supernatant as an initial reaction template. The initial reaction template was subjected to 10-fold serial dilution (10^−1^–10^−9^), and the reaction was carried out according to the optimized RT-LAMP reaction conditions. The reaction products were analyzed by LFD detection, calcein RT-LAMP assay and 1.2% agarose gel electrophoresis. Each test was repeated at least 3 times.

### RT-PCR

TRIzol reagent was used for RNA extraction, and the reverse transcription reaction system was 20 μL in volume: 4 μL of 5×AMV Buffer (5U, TaKaRa Biotechnology, Dalian, China), 2 μL of dNTPs (2.5 mM), 0.5 μL of RNase inhibitor (40 U, TaKaRa Biotechnology, Dalian, China), 1 μL of random primer (50 μM, TaKaRa Biotechnology, Dalian, China), 1 μL of AMV, and 10 μL (4.56ng) of RNA, supplemented with ddH_2_O to 20 μL. The cDNA obtained by reaction at 42°C for 1 h was used as a template, and SVA-F and SVA-R were used as primers for PCR. The PCR system volume was 25 μL: 2.5 μL of 10 × PCR Buffer, 2 μL of dNTPs (2.5 mM), 0.5 μL of SVA-F (10 μM), 0.5 μL of SVA-R (10 μM), 0.25 μL of Ex-Taq polymerase (5 U, TaKaRa Biotechnology, Dalian, China), and 2 μL of template, supplemented with ddH_2_O to 25 μL. The PCR conditions were predenaturation at 95°C for 3 min; denaturation at 95°C for 30 s, annealing at 56°C for 30 s, and extension at 72°C for 30 s, 30 cycles; final extension at 72°C for 10 min. The reaction product was analyzed by 1.2% agarose gel.

## Results

### Establishment of initial RT-LAMP-LFD detection method

**Fig 2:**
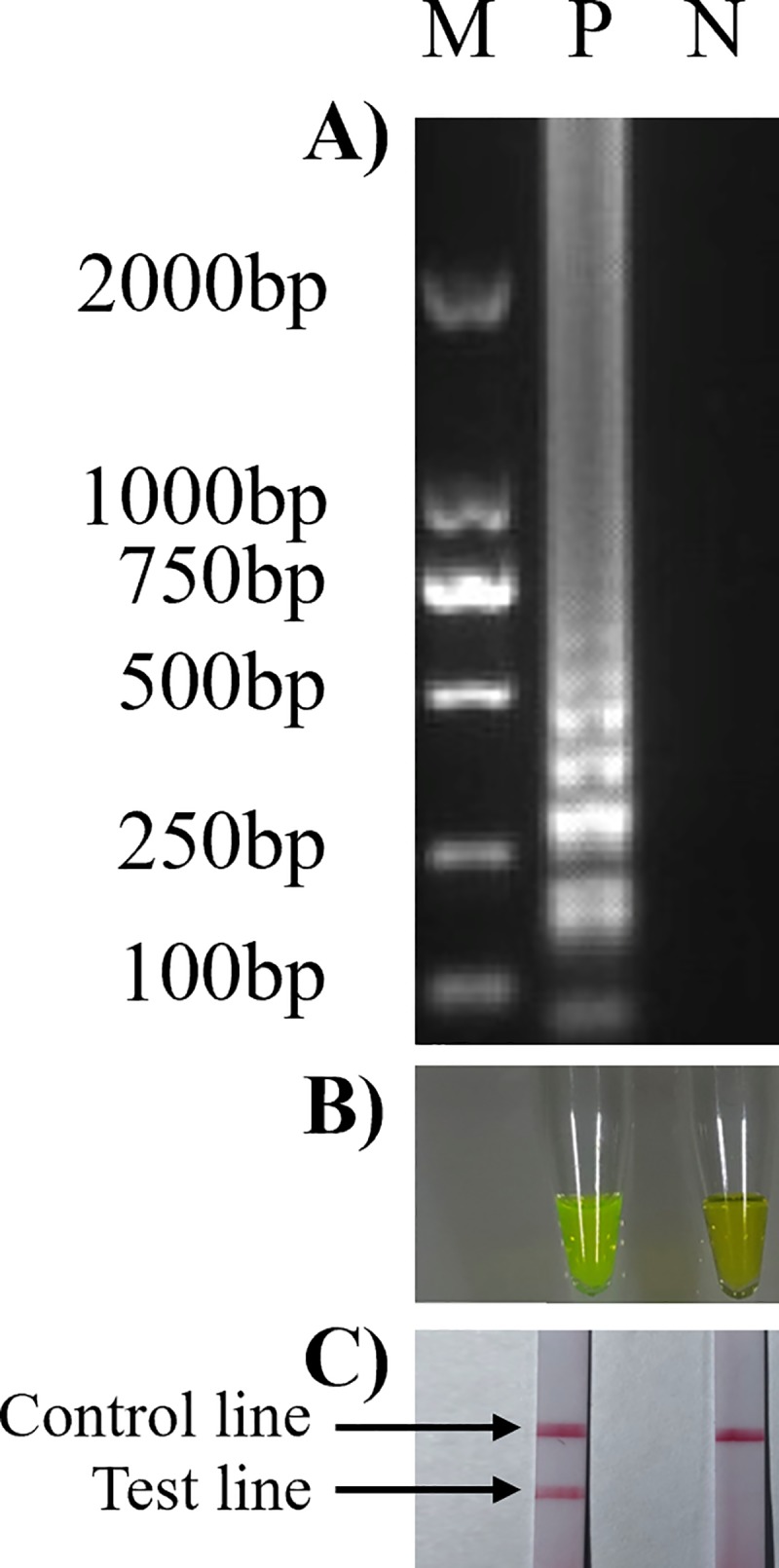
Establishment of SVA RT-LAMP-LFD detection method. A): RT-LAMP with gel electrophoresis detection: M: 2000 bp DNA marker, lane 1 for positive template (P), lane 2 for negative control (N); B): calcein RT-LAMP detection method: tube 1 for positive template (P), tube 2 for negative control (N); C): RT-LAMP-LFD detection method: strip 1 for positive template (P), strip 2 for negative control (N).

SVA HN16 strain RNA was used as a template for RT-LAMP reactions. The products were detected by 1.2% agarose gel electrophoresis, calcein assay and LFD assay to verify the feasibility of the RT-LAMP method. Agarose gel electrophoresis showed that the product resulting from the SVA-positive template had ladder-like bands and that the negative control had no bands (Fig 2A); the calcein method showed that the SVA-positive template product fluoresced green and that the negative control fluoresced orange (Fig 2B). The RT-LAMP-LFD results showed 2 red bands, one in the test line (T) and one in the quality control line (C), in the positive test group, while there was only one red strip (Fig 2C), in the quality control line (C), in the negative control group. The results show that the SVA RT-LAMP-LFD detection method is feasible.

### Optimization of SVA RT-LAMP reaction conditions

**Fig 3:**
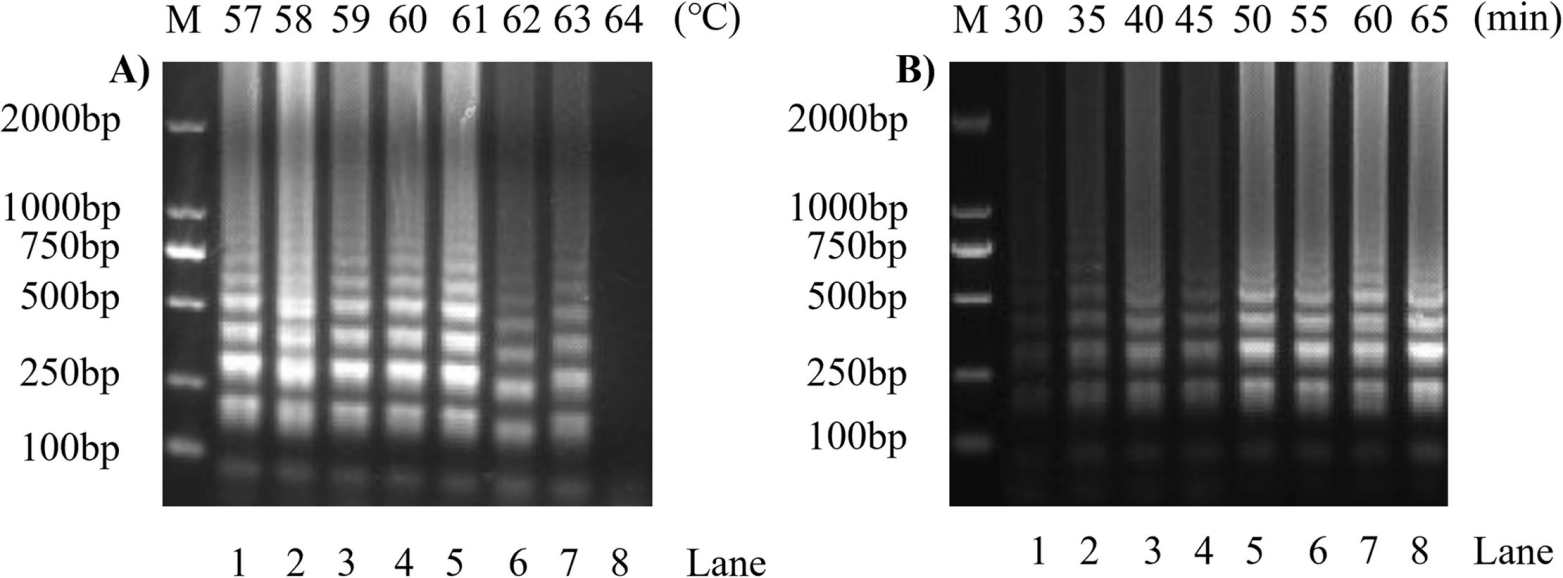
Optimization of SVA RT-LAMP reaction conditions. A): Reaction temperature optimization: M: 2000 bp DNA marker, lanes 1 to 8 represent 57°C, 58°C, 59°C, 60°C, 61°C, 62°C, 63°C, 64°C, for a gradient with 8 temperatures; B): Reaction time optimization: M: 2000 bp DNA marker, lanes 1 to 8 respectively represent reaction times of 30 min, 35 min, 40 min, 45 min, 50 min, 55 min, 60 min, and 65 min, for a gradient with 8 reaction times.

**Fig 4:**
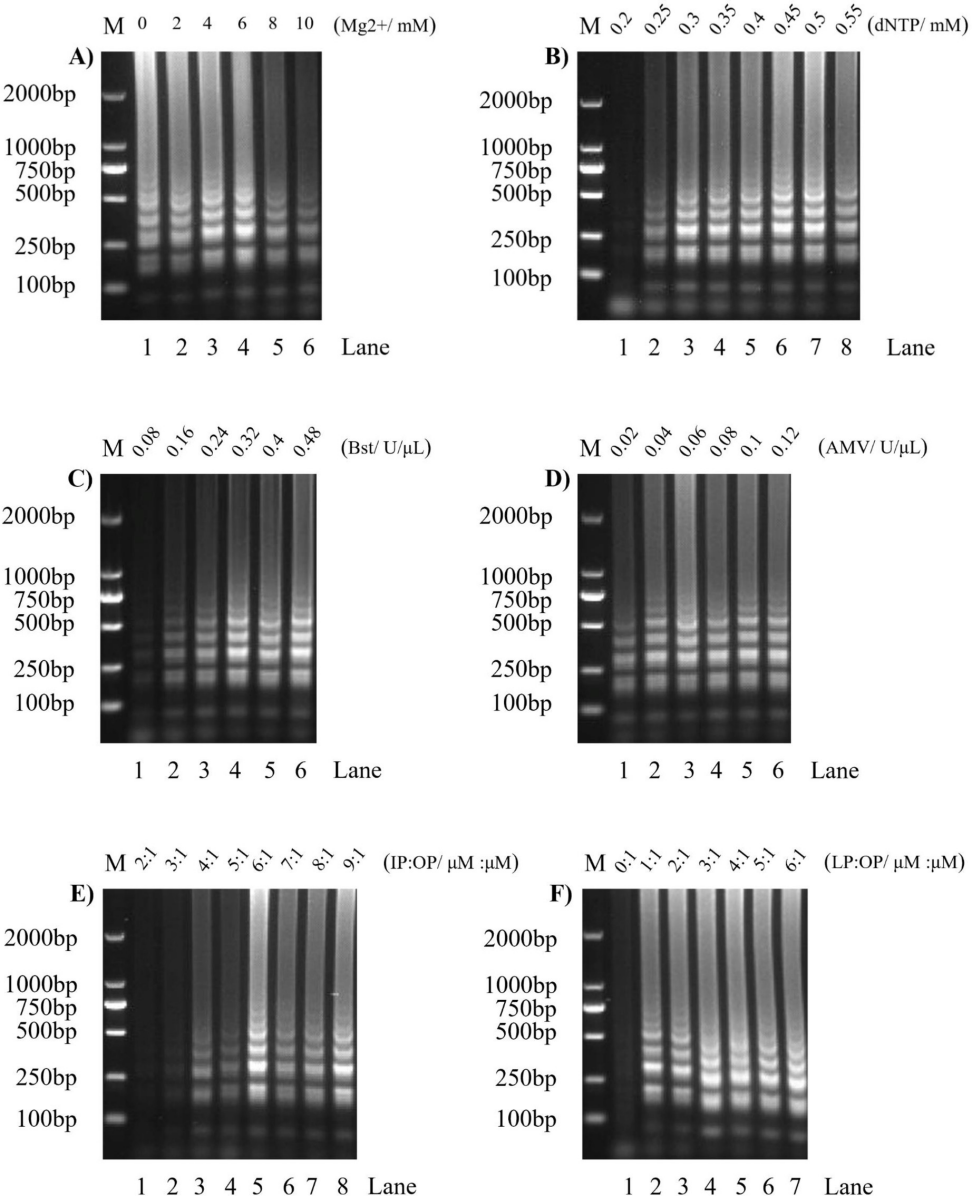
Optimization of SVA RT-LAMP reaction system. M: 2000 bp DNA marker; A): Mg2+ concentration optimization, lanes 1 to 6 represent 0 mM, 2 mM, 4 mM, 6 mM, 8 mM, and 10 mM, for 6 Mg2+ concentrations; B): dNTP concentration optimization, lanes 1 to 8 represent the 8 dNTP concentrations 0.2 mM, 0.25 mM, 0.3 mM, 0.35 mM, 0.4 mM, 0.45 mM, 0.5 mM, and 0.55 mM; C): Bst DNA polymerase concentration optimization, lanes 1 to 6 represent 0.08 U/μL, 0.16 U/μL, 0.24U/μL, 0.32U/μL, 0.4U/μL, and 0.48U/μL, for 6 Bst DNA Polymerase concentrations; D): AMV reverse transcriptase concentration optimization, lanes 1 to 6 represent the 6 AMV reverse transcriptase concentrations 0.02 U/μL, 0.04 U/μL, 0.06 U/μL, 0.08 U/μL, 0.1 U/μL, and 0.12 U/μL; E): optimization of internal primer to external primer ratio; in lanes 1 to 8, the ratio of the inner primer (IP) to the outer primer (OP) is 2:1, 3:1, 4:1, 5:1, 6:1, 7:1, 8:1, and 9:1; F): optimization of outer primer to external primer ratio; in lanes 1 to 7, the ratio of the loop primer (LP) to the outer primer (OP) is 0:1, 1:1, 2:1, 3:1, 4:1, 5:1, and 6:1.

In optimizing the SVA RT-LAMP reaction conditions, the RT-LAMP reaction was carried out at 8 different temperatures from 57–64°C, and the results of 1.2% agarose gel electrophoresis (Fig 3A) show that the enhanced band of lane 5 is the brightest, indicating that the optimum reaction temperature for the SVA RT-LAMP reaction is 61°C; the reaction time optimization results (Fig 3B) show that the brightness of the agarose gel electrophoresis band of the RT-LAMP reaction product was consistent after 50 min. Therefore, 50 min was selected as the optimal reaction time for the SVA RT-LAMP reaction. The optimization results for the reaction system are shown in Fig 4. The optimal reaction system of SVA RT-LAMP is 6 mM magnesium ions (Fig 4A), 0.5 mM dNTPs (Fig 4B). The optimal concentration of Bst DNA Polymerase was 0.32 U/μL (Fig 4C), the optimal AMV concentration was 0.06 U/μL (Fig 4D), and the optimal ratio of inner primer to external primer was 0.2 μM:0.12 μM (Fig 4E). The optimal ratio of loop primer to external primer was 0.04 μM:0.2 μM (Fig 4F).

### Specificity of SVA RT-LAMP-LFD detection method

**Fig 5:**
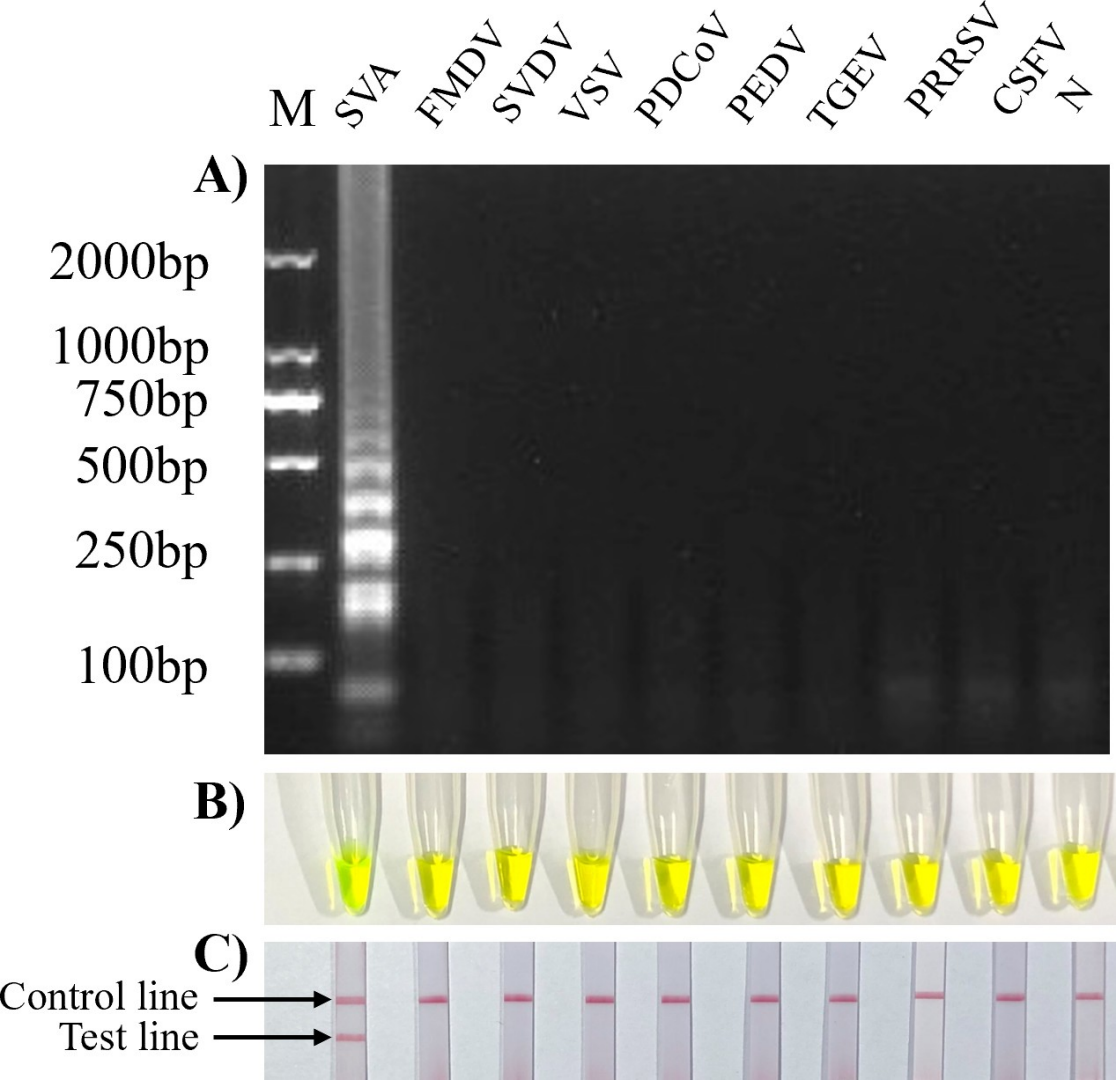
Specificity of SVA RT-LAMP-LFD detection method. M: 2000 bp DNA marker; lanes 1 to 10 are SVA, FMDV, SVDV, VSV, PDCoV, PEDV, TGEV, PRRSV, CSFV and negative control (N); A): specificity of RT-LAMP agarose gel electrophoresis assay; B): specificity of calcein RT-LAMP assay; and C): specificity of RT-LAMP-LFD assay.

To confirm the specificity of the SVA RT-LAMP-LFD detection method, the reaction was carried out using optimized reaction conditions. The viral RNA templates of SVA (HN16 strain), PDCoV (PDCoV/CHGD/2016 strain), PEDV (CH/GDGZ/2012 strain), TGEV (CN12 strain), PRRSV (JXA1 strain), the FMDV vaccine strain, the CSFV standard positive serum and positive plasmids of SVDV and VSV were separately detected by the RT-LAMP-LFD method and then analyzed by agarose gel electrophoresis and the calcein method. The results showed that SVA (HN16 strain) was positively amplified and the remaining pathogens were negatively amplified (Fig 5A–5C). The results indicated that the established SVA RT-LAMP-LFD detection method has good specificity.

### Sensitivity of SVA RT-LAMP-LFD detection method

**Fig 6:**
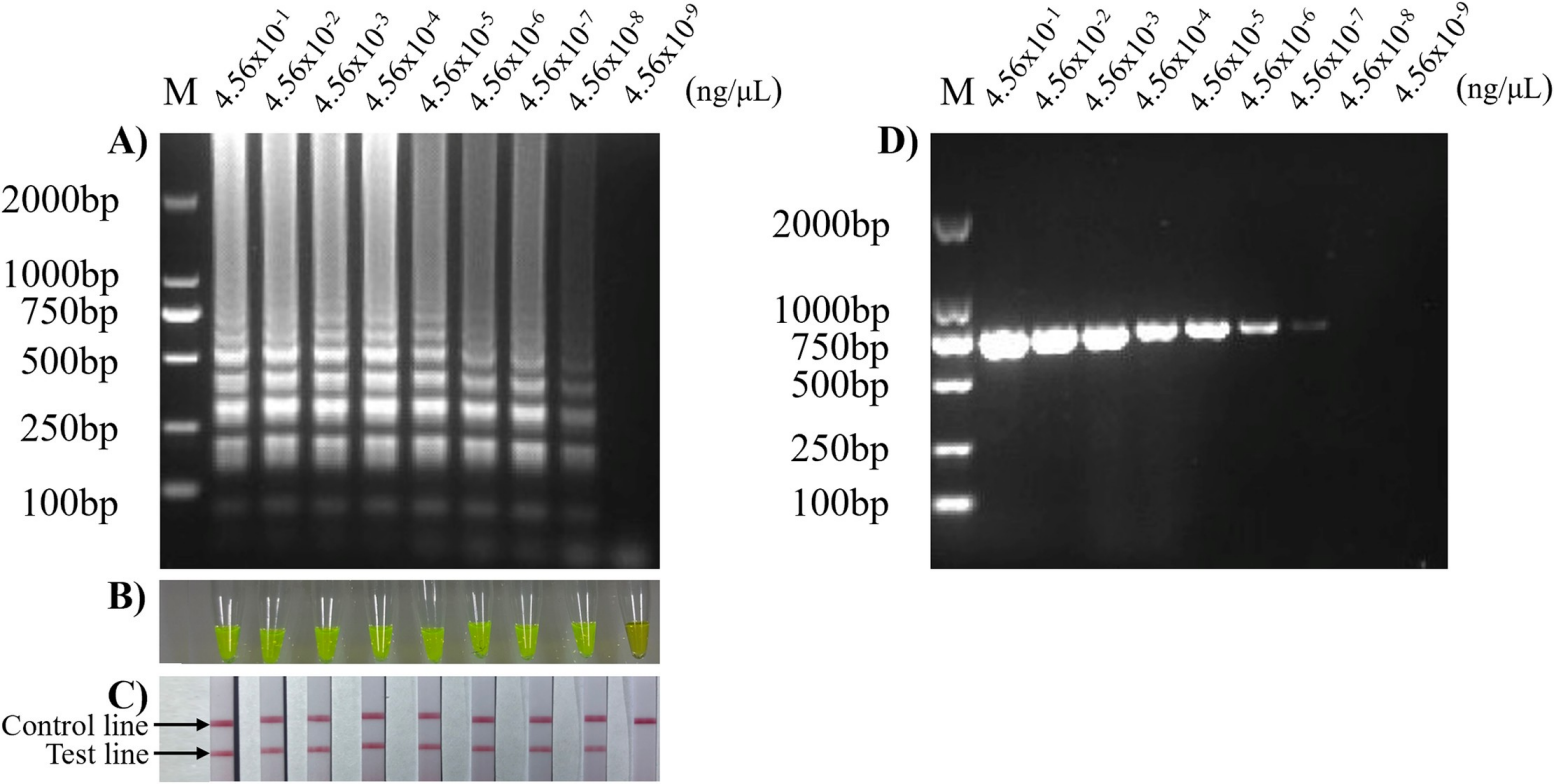
Sensitivity of SVA RT-LAMP-LFD detection method. 1: 4.56x10^-1^ ng/μL; 2: 4.56x10-2 ng/μL; 3: 4.56x10-3 ng/μL; 4: 4.56x10-4 ng/μL; 5: 4.56x10-5 ng/μL; 6: 4.56x10-6 ng/μL; 7: 4.56x10-7 ng/μL; 8: 4.56x10-8 ng/μL; and 9: 4.56x10-9 ng/μL; M: 2000 bp DNA marker; A): sensitivity of RT-LAMP agarose gel electrophoresis; B): sensitivity of calcein RT-LAMP under natural light; C): sensitivity of RT-LAMP-LFD; and D): sensitivity of RT-PCR.

To confirm the sensitivity of the SVA RT-LAMP-LFD detection method, the optimized reaction conditions were used for experiments, and a 10-fold gradient dilution of SVA HN16 genomic RNA was used as a template for the RT-LAMP reaction. The results showed that RT-LAMP agarose gel electrophoresis (Fig 6A), calcein RT-LAMP (Fig 6B) and RT-LAMP-LFD methods (Fig 6C) have the same sensitivity, and the lowest concentration of template detected was 4.5x10^-8^ ng/μL. However, this method is 10 times more sensitive than RT-PCR (4.5x10^-7^ ng/μL) (Fig 6D).

### Clinical application of SVA RT-LAMP-LFD detection method

Thirty-three seral samples clinically suspected of SVA infection were tested by RT-PCR and RT-LAMP-LFD. The results (Table 3) showed that 18 samples were positively amplified by RT-PCR and that the positive rate was 67.4%. When tested by the RT-LAMP-LFD method, 18 samples were positively amplified, and the positive rate was 67.4%. The coincidence rate of the two methods was 100%. The SVA RT-LAMP-LFD assay has the same clinical detection rate as the conventional RT-PCR assay.

**Table 3:** Clinical application of SVA RT-LAMP-LFD detection method.

Detection method	Positive samples	Negative samples	Positive rate /%	Coincidence rate of detection method /%
RT-PCR	18	15	67.4	100
RT-LAMP-LFD	18	15	67.4	

## Discussion

In this study, we first described a rapid detection method of RT-LAMP-LFD for SVA, which combined RT-LAMP with a LFD visualization strip to specifically detect SVA. The optimum LAMP reaction conditions were 61°C for 50 minutes. The LAMP products labeled with the probe were then hybridized in a closed lateral flow dipstick, and the results were visually observed by the naked eyes after 10–15 min. The entire process can be completed in less than 1h and requires no special equipment.

Since the development of the LAMP method[14], a series of LAMP and RT-LAMP assays have been applied to detect various of different pathogens infecting animals and human beings. The LAMP method were also combined with other simple methods, thus generating a variety of determination methods for results, including agarose gel electrophoresis, usage of dyes such as hydroxynaphthol blue or calcein[32], monitoring of accumulation of insoluble magnesium pyrophosphate by a turbidity device[33], and fluorescence dye detection by a fluorescence monitoring equipment (eg, a real-time PCR machine). However, these methods have higher requirements for hardware devices, and it is not easy to perform rapid detection at sites in the field. At present, various detection methods have been developed for the detection of SVA, such as real-time PCR[34], novel RNA-based in situ hybridization[12], enzyme-linked immunosorbent assay (ELISA)[13], etc. In addition, the LAMP detection method for SVA has been reported[35], but the method in this study required the Genie II instrument, which had high technical requirements for the experimenter and has not been widely used in the detection at sites in the field.

In this study, the existing SVA RT-LAMP detection method was improved by labeling the FIP and BIP with biotin and fluorescein isothiocyanate respectively, and lateral flow strips coated with biotin antibodies and isothiocyanate fluorescein antibodies were used for rapid detection of LAMP products. Compared with that of RT-PCR, the detection time established by this method is much shorter, and the detection sensitivity is 10 times higher, while the detection sensitivity of this method is equivalent with that of qRT-PCR[11]. Compared with the calcein RT-LAMP method, the method established in this experiment eliminates human error caused by visual observation of color, and the test is completed in a closed container, thus eliminating false-positive amplification caused by additional contamination of fluorescent dye in the RT-LAMP product. In some studies, only one labeled primer was used in the LAMP reaction[36, 37], and another labeled probe was added to the LAMP products to form a double-labeled detectable product, which easily caused contamination of the LAMP product. In our study, the two internal primers were labeled with biotin isothiocyanate and fluorescein isothiocyanate respectively. The results obtained from this and other studies indicate that the use of both labeled primers has no adverse effect on the LAMP reaction and it also showed that the method has the advantages of reducing contamination and easier operation [38–40].

In addition to the simple and rapid method of detecting RT-LAMP reaction products, this study also demonstrates that RT-LAMP amplification does not rely on strict constant temperature control and stringent time requirements. In temperature-optimized experiments, the amplification products of LAMP can be detected under 7 temperature gradients from 57°C to 63°C. The experimental results show that RT-LAMP-LFD does not require strictly controlled temperature. In the time-optimized experiment, the presence of the reaction product was clearly detected by agarose gel electrophoresis from 35 min, and saturation was observed after 50 min. Therefore, in order to save time in practical applications, the reaction time of LAMP can be controlled within a more flexible time range.

Primer design is the key to establishing RT-LAMP detection methods. Good primers can ensure the specificity of the detection method and the accuracy of the detection results. This experiment screened RT-LAMP primers designed based on the SVA 3D gene. The results indicate that the specific primers designed based on the SVA 3D gene have good specificity. Related studies have shown that the reaction conditions and systems of RT-LAMP, such as primer concentration, Mg^2+^ concentration and enzyme concentration, can directly affect the specificity and sensitivity of the detection[15]. Therefore, this experiment optimized the system and conditions of the established SVA RT-LAMP detection method, thus ensuring the high specificity and sensitivity of the detection method. In addition, the SVA RT-LAMP-LFD assay was found to have a higher clinical detection rate than RT-PCR in a clinical disease test.

In this study, a combination of RT-LAMP technology and LFD technology was applied to the detection of SVA and can now be used to analyze clinical samples such as SVA-infected tissues, vesicles and serum. The SVA RT-LAMP-LFD assay established in this trial aims to become a rapid and convenient diagnostic method for normal or suspected SVA-infected pigs at sites in the field. Its application value mainly lies in the initial diagnosis of SVA-infected pigs, the rapid screening of SVA in import and export trade, and the removal of SVA from pig farms. SVA RT-LAMP-LFD is a promising new tool for diagnosing SVA infection and is expected to have good application prospects.

## Supporting information

S1 Fig The alignment results of 3D regions base on the thirty-one full-length SVA sequences. The figure shows the location of RT-LAMP primer binding sites within 3D genes.
(TIF)
