# Supplementary figures and images for: Rapid and sensitive detection of Senecavirus A by reverse transcription loop-mediated isothermal amplification combined with a lateral flow dipstick method
Source: PLoS One. 2019 May 2;14(5):e0216245. doi: 10.1371/journal.pone.0216245 (PMC6497277; doi:10.1371/journal.pone.0216245)

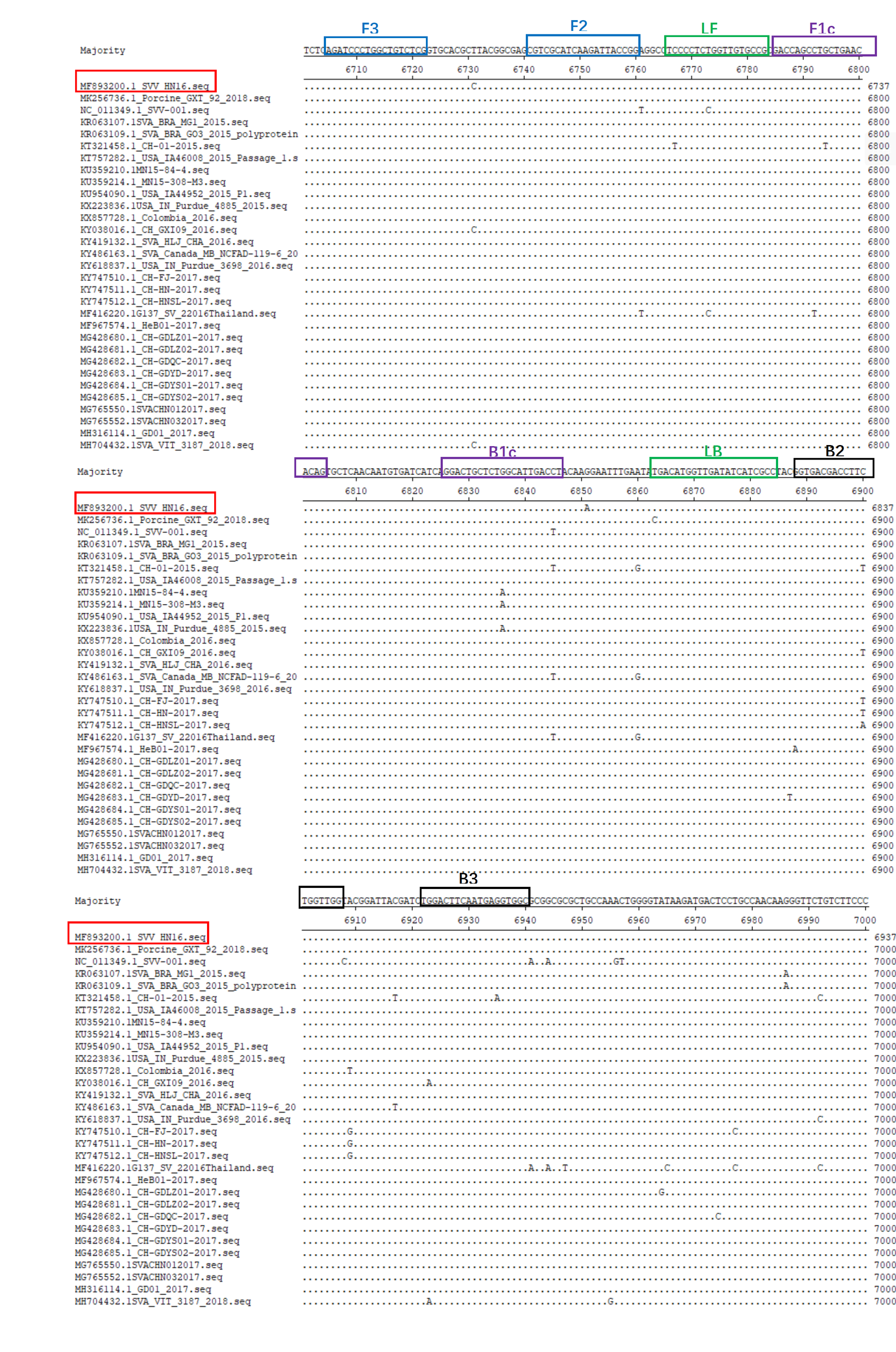

Supplement: S1 Fig — The figure shows the location of RT-LAMP primer binding sites within 3D genes. (TIF) [file pone.0216245.s001.tif]
